# PTH-to-tumor-volume ratio as a potential exploratory marker in parathyroid carcinoma: a retrospective cohort study

**DOI:** 10.1007/s13304-026-02868-4

**Published:** 2026-10-16

**Authors:** Magdalena Kwapisz, Jacek Gałczyński, Elwira Bakuła Zalewska, Agnieszka Żyłka, Piotr Góralski, Aleksandra Dworakowska, Marek Dedecjus

**Affiliations:** 1 Department of Endocrine Oncology and Nuclear Medicine, Maria Sklodowska-Curie National Research Institute of Oncology, Roentgena 5 Str, Warsaw, 02-781 Poland; 2 Department of Pathology, Maria Sklodowska-Curie National Research Institute of Oncology, Warsaw, 02-781 Poland

**Keywords:** Hyperparathyroidism, Primary, Parathyroid Neoplasms, Parathyroid Carcinoma, Parathyroid Hormone, Diagnostic Techniques, Endocrine

## Abstract

**Background:**

Parathyroid carcinoma is a rare endocrine malignancy that remains difficult to distinguish preoperatively from benign causes of primary hyperparathyroidism due to overlapping clinical and biochemical features. This study evaluates the serum parathyroid hormone (PTH)-to-tumor volume ratio as a potential parameter for differentiating parathyroid carcinoma from benign parathyroid lesions.

**Methods:**

A retrospective analysis was conducted on 344 patients who underwent surgery for primary hyperparathyroidism between 2017 and 2024. Patients were divided into carcinoma (*n* = 8) and benign lesion groups (*n* = 336). Clinical, biochemical, and ultrasound-derived tumor volume data were analyzed and compared between groups.

**Results:**

Patients with parathyroid carcinoma had significantly higher PTH levels than those with benign lesions (median, 659 vs. 136.4 pg/mL; *p* = 0.002) and markedly larger tumor volumes (median, 10.14 vs. 0.69 ml; *p* < 0.001). Despite higher absolute PTH concentrations, the PTH-to-tumor volume ratio was significantly lower in the carcinoma group (median 89.7 vs. 214.56; *p* = 0.011). In logistic regression analysis, a lower PTH-to-tumor volume ratio was associated with an increased likelihood of parathyroid carcinoma (OR, 0.990; 95% CI: 0.896–0.993; *p* = 0.04).

**Conclusions:**

In this cohort, parathyroid carcinoma was characterized by a substantially greater tumor burden and higher circulating PTH concentrations, while the PTH-to-tumor-volume ratio was lower than in benign parathyroid lesions. The observed difference in the ratio is hypothesis-generating and warrants confirmation in larger, multicenter studies. Given the limited number of carcinoma cases, its role in clinical decision-making remains to be established.

**Graphical abstract:**

**Figure:**
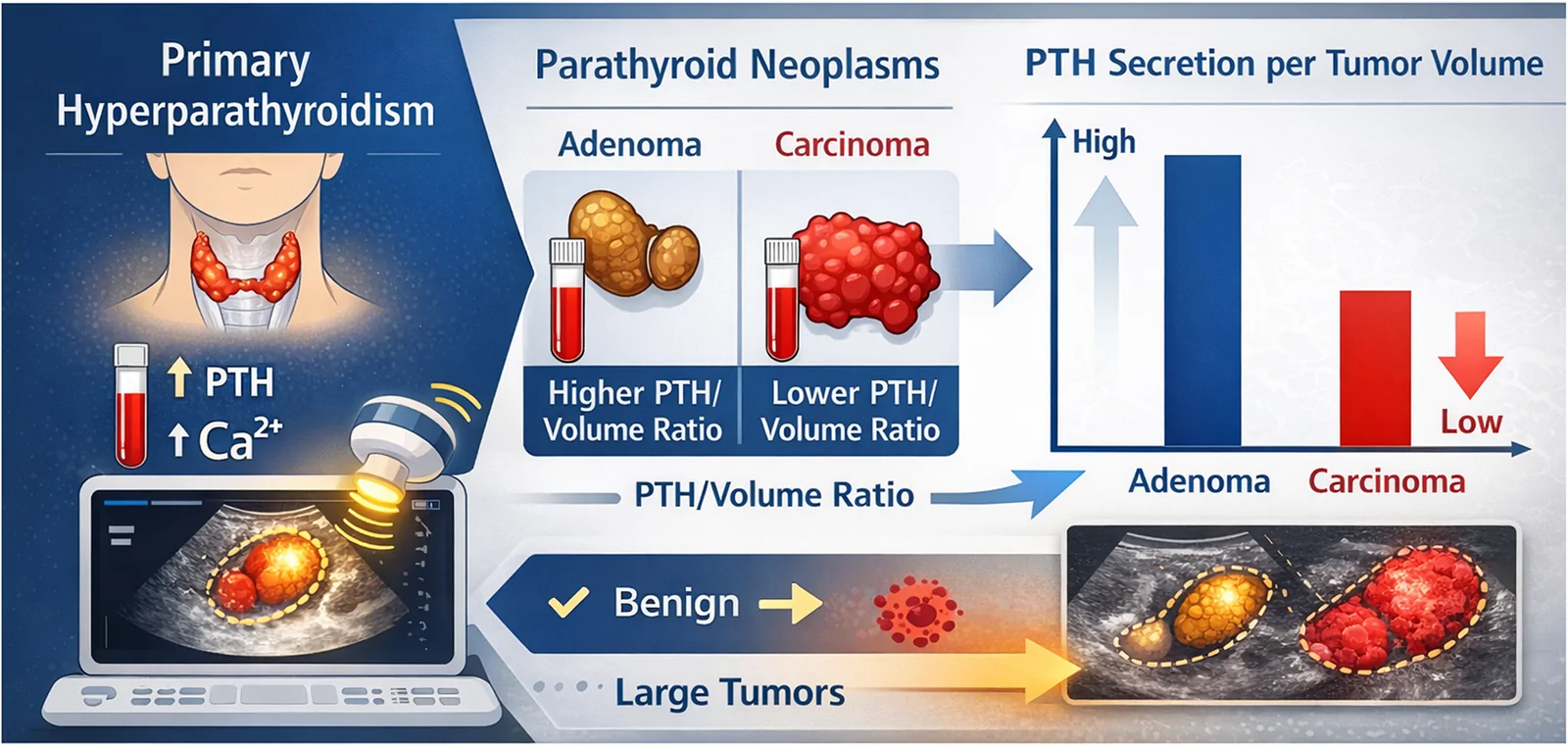

## Introduction

Parathyroid carcinoma (PC) is an exceptionally rare endocrine malignancy, accounting for less than 1% of cases of primary hyperparathyroidism (PHPT) and less than 0.005% of all malignancies worldwide, which places it at the margins of both endocrinology and oncology [1]. Despite its rarity, PC is clinically important because it is frequently associated with severe hypercalcemia and markedly elevated parathyroid hormone (PTH) concentrations, which may lead to significant morbidity and, in advanced cases, mortality [1–3]. Parathyroid carcinoma most commonly presents in middle age, with no distinct sex predilection, and is characterized by a tendency toward local invasion and metastatic spread [4–6]. Although the disease generally follows a relatively slow clinical course, lymph node metastases occur in approximately 5% of patients and distant metastases in approximately 2% [4]. Nevertheless, PC may demonstrate aggressive behavior through direct invasion of adjacent structures and lymphatic or hematogenous dissemination, with the lungs, bones, and liver being the most commonly affected distant sites [6]. The reported 5-year overall survival rate is approximately 82.7%; however, it decreases to 72.2% among patients treated with radiation therapy alone. Importantly, mortality is most commonly related to severe, uncontrolled hypercalcemia rather than direct organ damage caused by tumor burden [2].

Accurate diagnosis is particularly important because the initial surgical approach has a major impact on disease control. When PC is suspected, primary radical *en-bloc* resection is generally considered the preferred surgical strategy and is associated with lower recurrence rates than initial limited parathyroidectomy followed by delayed reoperation [7–10]. Therefore, identifying patients at increased risk of carcinoma before or during the initial operation remains an important clinical objective. Preoperative diagnosis of PC remains challenging because its clinical and biochemical features substantially overlap with those of benign parathyroid disease. Patients with carcinoma often present with markedly elevated serum PTH and calcium concentrations and larger tumors than patients with benign parathyroid adenomas; however, these parameters alone cannot reliably distinguish malignant from benign lesions [11, 12]. Similarly, conventional imaging modalities, including ultrasonography, computed tomography, and magnetic resonance imaging, are primarily used for tumor localization and have limited ability to distinguish malignant from benign parathyroid lesions [13]. Fine-needle aspiration biopsy (FNAB) is generally discouraged because of its limited diagnostic value and the potential risk of tumor cell dissemination. Furthermore, the distinction between atypical parathyroid tumors and carcinoma may remain difficult even on histopathological examination [13]. Consequently, the diagnosis of PC is frequently established only after surgery, potentially limiting the opportunity to perform an appropriate primary radical surgical procedure [11, 12].

Recent observations suggest that the relationship between hormonal secretion and tumor volume may provide additional information for differentiating malignant from benign parathyroid lesions [14]. Although parathyroid carcinomas are often characterized by substantial tumor size and markedly elevated PTH concentrations, the increase in hormone secretion may not be proportional to tumor volume. This may indicate a lower secretory efficiency per unit of tumor tissue compared with benign parathyroid adenomas. Therefore, evaluating PTH concentration in relation to tumor volume may provide additional information beyond that obtained from either parameter alone. Based on this concept, the serum PTH-to-tumor volume ratio may serve as an integrated marker of hormonal secretion relative to tumor burden. A lower ratio could reflect the potentially reduced secretory efficiency of parathyroid carcinoma and may help distinguish malignant from benign parathyroid lesions. Such an approach could complement conventional biochemical and radiological parameters and potentially improve preoperative risk stratification in patients with PHPT. The aim of this study was to evaluate whether the serum PTH-to-tumor volume ratio could serve as a potential marker of reduced secretory efficiency in parathyroid carcinoma and to determine its utility in differentiating malignant from benign parathyroid lesions in patients with primary hyperparathyroidism.

## Methods

This retrospective analysis included all consecutive patients who underwent surgical treatment for primary hyperparathyroidism between January 2017 and December 2024 at the Maria Sklodowska-Curie National Research Institute of Oncology in Warsaw, Poland. During the study period, 367 patients underwent surgery for hyperparathyroidism and were initially screened for eligibility.

Patients were eligible for inclusion if they met all of the following criteria:

- confirmed diagnosis of primary hyperparathyroidism;
- surgical treatment of a parathyroid lesion;
- availability of preoperative biochemical, ultrasound, and histopathological data required for the analysis.

Patients were excluded for the following reasons: parathyromatosis (*n* = 1), multiglandular disease (*n* = 5), previous unsuccessful parathyroid surgery (*n* = 2), secondary hyperparathyroidism in patients undergoing hemodialysis before renal transplantation (*n* = 4), lack of a preoperative ultrasound measurement of the parathyroid lesion (*n* = 4), missing histopathological data (*n* = 5), and incomplete clinical data (*n* = 2). Thus, 23 patients were excluded, resulting in a final study population of 344 patients.

**Fig. 1:**
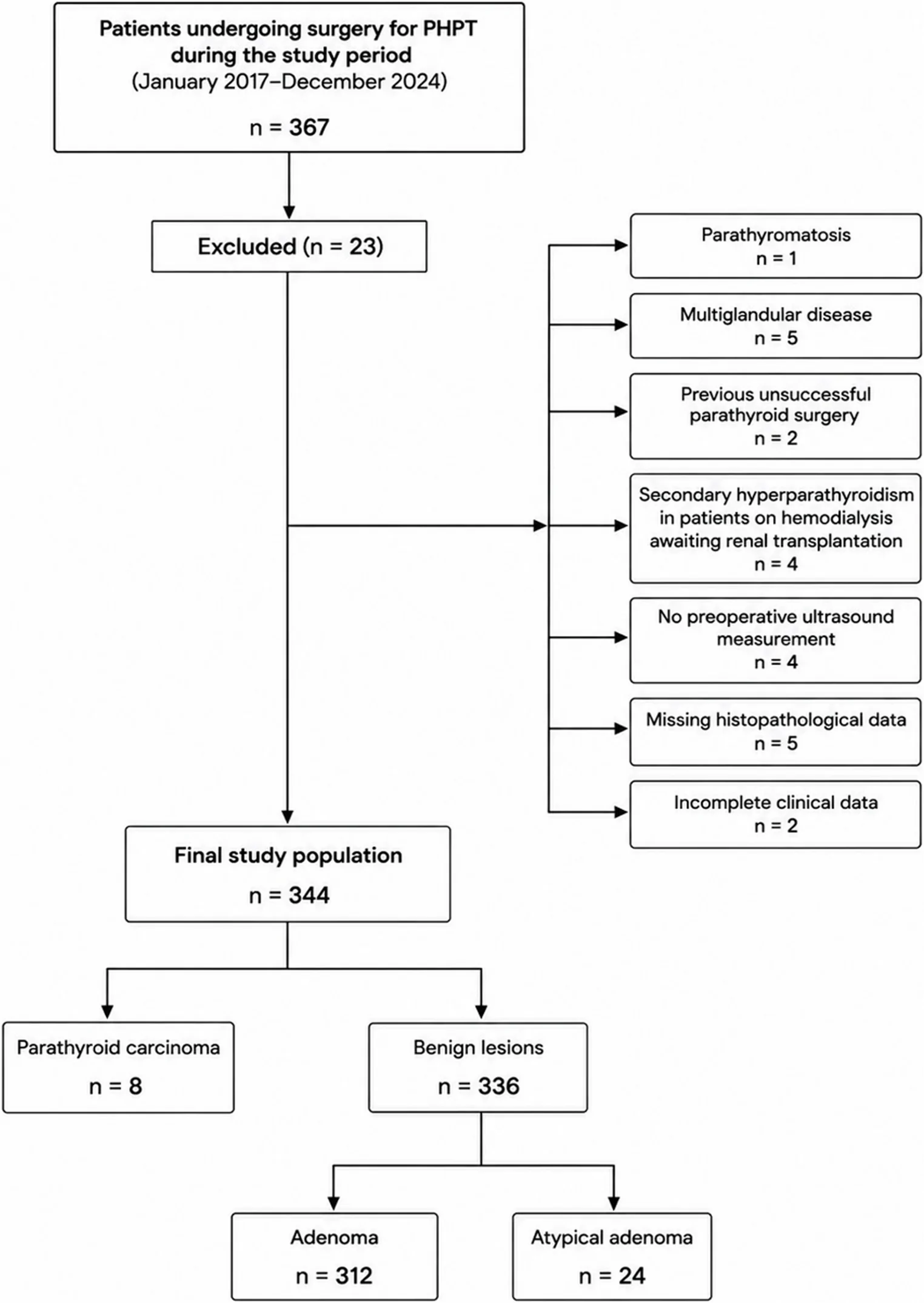
Flow diagram of patient selection and study population

Importantly, multiglandular disease was defined as the presence of more than one enlarged parathyroid gland identified during diagnostic evaluation and/or surgery, rather than on the basis of a specific genetic syndrome. Patients with multiple endocrine neoplasia (MEN) were therefore not excluded solely on the basis of the underlying syndrome. Patients with MEN in whom a single enlarged parathyroid gland was identified and surgically removed were eligible for inclusion. Among the 344 patients included in the final analysis, 8 patients were diagnosed with parathyroid carcinoma and 336 with benign parathyroid lesions. The benign group included parathyroid adenomas and atypical adenomas (Fig. 1).

Based on the final histopathological diagnosis, patients were divided into two main groups: those with parathyroid carcinoma and those with benign lesions, including parathyroid adenoma and atypical parathyroid adenoma. The analyzed variables included demographic data (age and sex), biochemical parameters (PTH levels and serum calcium concentration), and morphological parameters, particularly tumor volume measured on preoperative ultrasound. Histopathological evaluation was performed by a single experienced pathologist and was conducted in accordance with the World Health Organization (WHO) classification criteria. Preoperative ultrasound examinations were carried out by a specialist with over 10 years of experience, certified by the Polish Ultrasound Society in thyroid and parathyroid ultrasonography. At the time of ultrasound examination, the examiner was blinded to the patients’ biochemical parameters and subsequent final histopathological diagnoses. The ultrasound findings and measurements were subsequently retrospectively retrieved from the patients’ medical records and included in the analysis. Laboratory tests were performed in the hospital’s clinical laboratory in accordance with current standardization protocols. Serum PTH levels were measured using a second-generation (intact) electrochemiluminescence immunoassay (Elecsys PTH, Roche Diagnostics) on an automated analyzer.

At the stage of statistical analysis, data were presented as mean ± standard deviation (SD) for normally distributed variables and as median (interquartile range, IQR) for non-normally distributed variables. Parametric variables were compared using Student’s t-test, whereas nonparametric variables were analyzed using the Mann–Whitney U test. Categorical variables were compared using the chi-square test or Fisher’s exact test, as appropriate. Intergroup comparisons were performed using the Mann–Whitney U test. Logistic regression analysis was conducted to assess potential predictors of parathyroid carcinoma. Odds ratios (ORs) with 95% confidence intervals (CIs) were calculated for tumor volume and the PTH-to-tumor-volume ratio. All statistical analyses were performed using IBM SPSS Statistics, version 27.0 (IBM Corp., Armonk, NY, USA). A p-value of < 0.05 was considered statistically significant.

## Results

### Baseline characteristics

A total of 344 patients who underwent surgery for primary hyperparathyroidism (PHPT) were included in the analysis. The study population comprised 56 male and 288 female patients, with a mean age of 56.15 ± 13.88 years (range, 18–88 years). Parathyroid carcinoma (PC) was diagnosed in 8 patients (Group 1), while 336 patients had benign parathyroid lesions (Group 2). Thus, PC accounted for 2.3% of the analyzed cohort, representing a higher proportion than that reported in the general PHPT population. This difference may be attributable to the tertiary referral nature of our institution, which serves as a referral center for patients with suspected or confirmed parathyroid malignancy and may therefore result in referral bias and enrichment of the study population with patients at higher risk of PC. To ensure diagnostic accuracy, all cases classified as PC underwent independent histopathological review by a second pathologist, followed by discussion and confirmation at a multidisciplinary pathology board.

In the benign group, histopathological examination identified 312 parathyroid adenomas and 24 atypical adenomas. In the carcinoma group, all 8 tumors met the WHO histopathological criteria for parathyroid carcinoma [15]. Unequivocal vascular invasion was present in 5 cases, while in 3 cases only rare intravascular tumor cells were identified, without definite evidence of vascular invasion. In these three cases, the diagnosis of carcinoma was supported by other established histopathological criteria of malignancy. Capsular invasion was present in 7 cases (87.5%), perineural invasion was not identified, and invasion of adjacent structures was observed in 3 cases. No lymph node involvement or distant metastases were documented in the available cases. Based on Schulte’s Staging System [16], 3 tumors were classified as low-risk and 5 as high-risk. No disease recurrence was documented during the available follow-up period. The clinical and histopathological characteristics of the study groups are summarized in Table 1.

**Table 1:** Detailed clinical, biochemical, and histopathological characteristics of the study cohort

Variable	Parathyroid carcinoma (*n* = 8)	Benign lesions (*n* = 336)	*p*-value
Demographics			
Age (years), mean ± SD	50.75 ± 9.76	56.28 ± 13.94	0.266
Sex (female/male), n	07/01	281 / 55	NS
Biochemical parameters			
Serum calcium (mmol/L), median (IQR)	3.03 (2.88–3.15)	2.82 (2.70–2.96)	0.047
PTH (pg/mL), median (IQR)	659 (202.5–2580)	136.4 (99.29–227)	0.002
Morphological parameters			
Tumor volume (mL), median (IQR)	10.14 (7.11–13.42)	0.69 (0.31–1.41)	< 0.001
Derived parameters			
PTH/tumor volume ratio, median (IQR)	89.7 (50.15–156.13)	214.56 (114.34–399.59)	0.011
Histopathological characteristics			
Parathyroid adenoma, n (%)	—	312 (92.9%)	—
Atypical adenoma, n (%)	—	24 (7.1%)	—
Carcinoma staging (Schulte classification)			
Low-risk, n (%)	3 (37.5%)	—	—
High-risk, n (%)	5 (62.5%)	—	—

*SD* standard deviation, *IQR* interquartile range, *PTH* parathyroid hormone, *NS* not significant; P-values were calculated using Student’s t-test, Mann–Whitney U test, or chi-square/Fisher’s exact test, as appropriate

The distribution of sex and age at the time of surgery was compared between the groups. In Group 1, there were 7 females and 1 male, while Group 2 comprised 281 females and 55 males. The mean age at surgery was 50.75 ± 9.76 years in Group 1 and 56.28 ± 13.94 years in Group 2. The difference in mean age between the groups was 5.53 years and was not statistically significant (t(342) = − 1.12, *p* = 0.266).

### Biochemical characteristics

Serum calcium and PTH levels were assessed on the day prior to surgery. Serum calcium levels were significantly higher in patients with PC than in those with benign lesions, with a median of 3.03 mmol/L (IQR: 2.88–3.15) in Group 1 compared with 2.82 mmol/L (IQR: 2.70–2.96) in Group 2 (U = 757.5, *p* = 0.047). Notably, these measurements were obtained upon hospital admission for surgery, following pharmacological management of hypercalcemia aimed at reducing the risk of hypercalcemic crisis. Despite this preoperative treatment, serum calcium levels remained significantly higher in patients with PC. Preoperative serum PTH levels were also significantly higher in the carcinoma group, with a median of 659 pg/mL (IQR: 202.5–2580) compared with 136.4 pg/mL (IQR: 99.29–227) in the benign group (U = 461.0, *p* = 0.002).

### Tumor volume

Preoperative ultrasound examination was used to assess tumor morphology and size. Tumor volume was calculated from three-dimensional measurements using the ellipsoid formula (π/6 × *a* × *b* × *c*), where *a*, *b*, and *c* represented the three orthogonal tumor dimensions. Tumor volume was significantly greater in the carcinoma group than in the benign lesion group. The median tumor volume was 10.14 mL (IQR: 7.11–13.42) in Group 1 and 0.69 mL (IQR: 0.31–1.41) in Group 2 (U = 136.0, *p* < 0.001).

### PTH-to-tumor-volume ratio

**Fig. 2:**
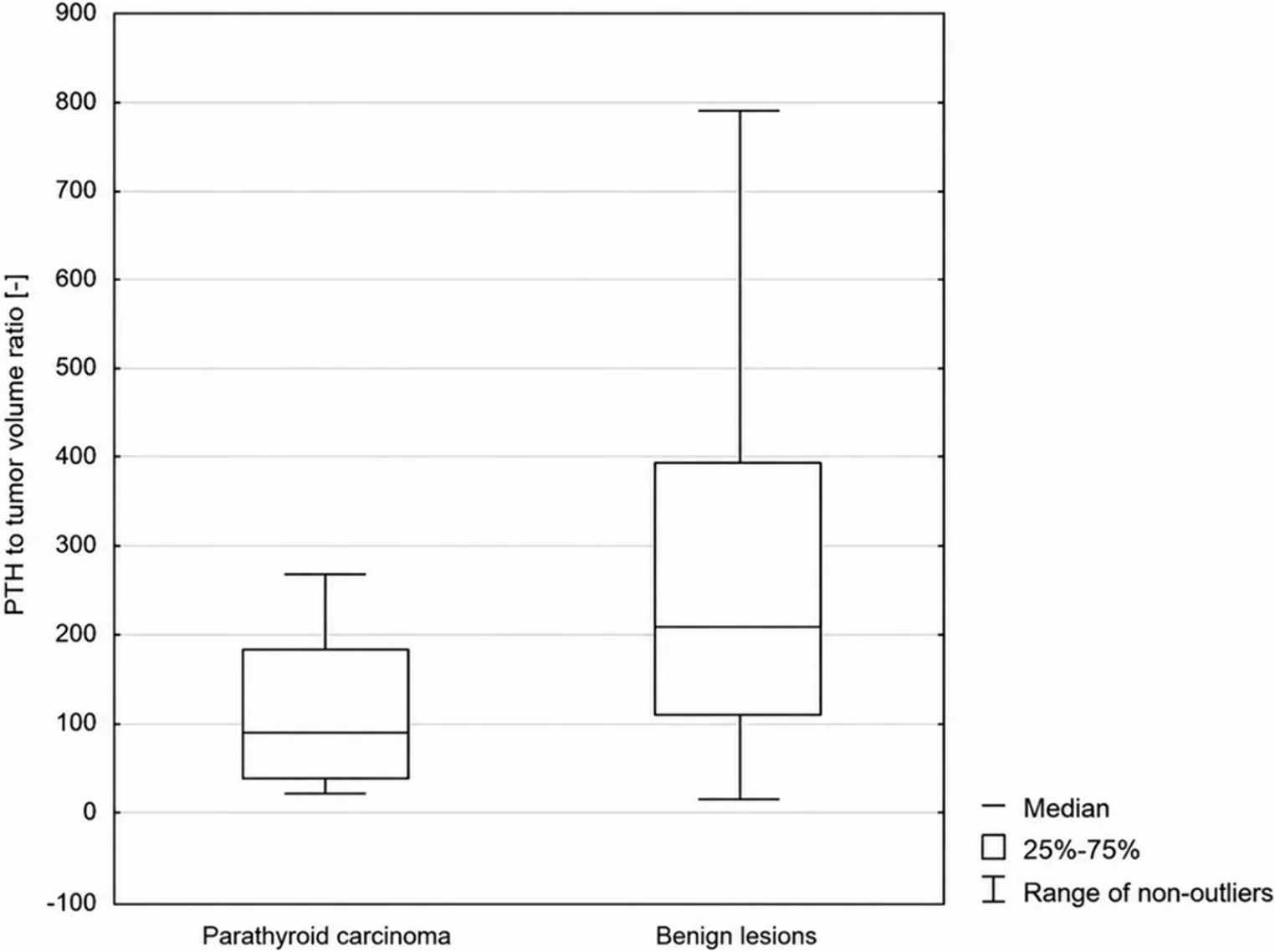
Comparison of the PTH-to-tumor-volume ratio between parathyroid carcinoma and benign lesions. Box-and-whisker plots show median and interquartile range, with individual observations overlaid. The PTH-to-tumor-volume ratio was significantly lower in carcinoma compared with benign lesions (*p* = 0.011), consistent with reduced secretory efficiency

The serum PTH-to-tumor-volume ratio was calculated for each patient to assess the relationship between hormonal secretion and tumor volume. Despite higher absolute PTH concentrations and substantially larger tumor volumes in patients with PC, the PTH-to-tumor-volume ratio was significantly lower in the carcinoma group than in the benign group. The median ratio was 89.7 (IQR: 50.15–156.13) in Group 1 compared with 214.56 (IQR: 114.34–399.59) in Group 2 (U = 1672.0, *p* = 0.011; Fig. 2).

**Fig. 3:**
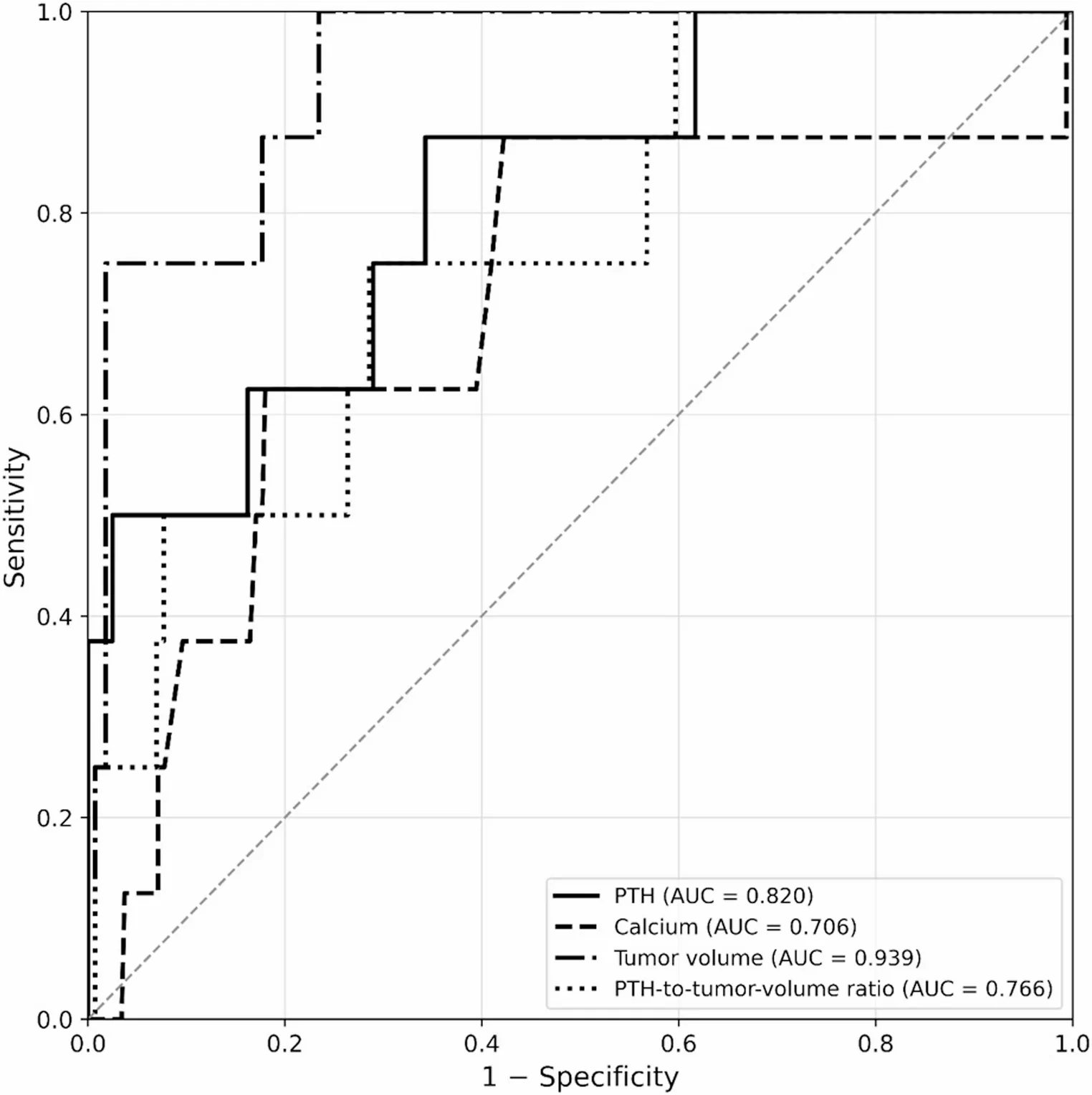
ROC curves for preoperative predictors of parathyroid carcinoma

Receiver operating characteristic (ROC) curve analysis was performed to evaluate and compare the diagnostic performance of preoperative serum PTH, serum calcium, tumor volume, and the PTH-to-tumor-volume ratio for differentiating parathyroid carcinoma from benign parathyroid lesions (Fig. 3). Parathyroid carcinoma was considered the positive outcome. The optimal cut-off value for each parameter was determined using the Youden index (sensitivity + specificity − 1). The area under the ROC curve (AUC) and its 95% confidence interval (CI) were calculated using the DeLong method, while 95% CIs for sensitivity, specificity, positive predictive value (PPV), and negative predictive value (NPV) were calculated using the exact binomial method.

Serum PTH demonstrated good discriminatory performance, with an AUC of 0.821 (95% CI, 0.665–0.977). The optimal cut-off value was ≥ 184.5 pg/mL, yielding a sensitivity of 87.5% (95% CI, 47.3–99.7%) and a specificity of 65.8% (95% CI, 60.4–71.0%). The corresponding PPV was 5.9% (95% CI, 2.4–11.8%), whereas the NPV was 99.5% (95% CI, 97.4–100%).

Serum calcium demonstrated lower discriminatory performance, with an AUC of 0.704 (95% CI, 0.483–0.924). The optimal cut-off value was ≥ 2.87 mmol/L, corresponding to a sensitivity of 87.5% (95% CI, 47.3–99.7%) and a specificity of 57.7% (95% CI, 52.1–63.1%). The PPV was 4.8% (95% CI, 2.0–9.7%), while the NPV was 99.5% (95% CI, 97.1–100%).

Tumor volume demonstrated the highest discriminatory performance among the individual parameters, with an AUC of 0.940 (95% CI, 0.876–1.000). The optimal cut-off value was ≥ 1.54 mL, yielding a sensitivity of 100.0% (95% CI, 63.1–100%) and a specificity of 76.9% (95% CI, 71.5–81.7%). The PPV was 11.0% (95% CI, 4.9–20.5%), while the NPV was 100.0% (95% CI, 98.3–100%).

The PTH-to-tumor-volume ratio demonstrated an AUC of 0.765 (95% CI, 0.596–0.935). As lower values of the ratio were associated with parathyroid carcinoma, decreasing ratio values were considered indicative of a higher probability of carcinoma. The optimal cut-off value was ≤ 126.30, corresponding to a sensitivity of 75.0% (95% CI, 34.9–96.8%) and a specificity of 71.6% (95% CI, 65.9–76.9%). The PPV was 7.1% (95% CI, 2.7–14.9%), and the NPV was 99.0% (95% CI, 96.4–99.9%).

Pairwise comparison of the AUCs using the paired DeLong test showed that tumor volume had significantly greater discriminatory performance than serum PTH (AUC, 0.940 vs. 0.820; *p* = 0.044) and serum calcium (AUC, 0.939 vs. 0.702; *p* = 0.046). No statistically significant difference was observed between PTH and calcium (*p* = 0.376). Similarly, the AUC of the PTH-to-tumor-volume ratio did not differ significantly from that of PTH (*p* = 0.709), calcium (*p* = 0.678), or tumor volume (*p* = 0.071).

### Logistic regression analysis

Univariable logistic regression analysis was performed to further evaluate the association between individual preoperative parameters and the likelihood of parathyroid carcinoma. Serum PTH was significantly associated with PC, with an odds ratio (OR) of 1.231 per 100 pg/mL increase (95% CI, 1.124–1.349; *p* < 0.001). Tumor volume was also significantly associated with PC, with an OR of 1.299 per 1 mL increase (95% CI, 1.159–1.456; *p* < 0.001). In contrast, serum calcium was not significantly associated with PC (OR, 0.9997 per 0.1 mmol/L increase; 95% CI, 0.993–1.006; *p* = 0.922).

The PTH-to-tumor-volume ratio showed a borderline association with PC in univariable analysis. A 100-unit increase in the ratio was associated with lower odds of PC (OR, 0.421; 95% CI, 0.173–1.028; *p* = 0.058), although this association did not reach conventional statistical significance.

In a multivariable logistic regression model including serum PTH, calcium, and tumor volume, both PTH and tumor volume remained independently associated with PC. Each 1 pg/mL increase in PTH was associated with an OR of 1.002 (95% CI, 1.000–1.003; *p* = 0.029), while each 1 mL increase in tumor volume was associated with an OR of 1.212 (95% CI, 1.051–1.398; *p* = 0.008). Serum calcium was not independently associated with PC (OR, 0.278; 95% CI, 0.020–3.828; *p* = 0.339).

A model including PTH and tumor volume demonstrated that both parameters remained statistically significant predictors of PC. Tumor volume was associated with an OR of 1.197 per 1 mL increase (95% CI, 1.044–1.373; *p* = 0.010), while PTH was associated with an OR of 1.001 per 1 pg/mL increase (95% CI, 1.000–1.002; *p* = 0.039).

In a separate logistic regression model including tumor volume and the PTH-to-tumor-volume ratio, tumor volume remained significantly associated with PC (OR, 1.263 per 1 mL increase; 95% CI, 1.116–1.429; *p* < 0.001). The PTH-to-tumor-volume ratio was inversely associated with the likelihood of PC, with each one-unit increase in the ratio corresponding to an approximately 1% decrease in the odds of parathyroid carcinoma (OR, 0.990; 95% CI, 0.896–0.993; *p* = 0.04). Conversely, a decrease in the PTH-to-tumor-volume ratio was associated with an approximately 1% increase in the odds of PC.

Overall, tumor volume demonstrated the strongest discriminatory performance in ROC analysis and remained a significant predictor of PC in logistic regression models. Although the PTH-to-tumor-volume ratio was significantly lower in patients with parathyroid carcinoma in the comparative analysis and demonstrated moderate discriminatory performance in ROC analysis (AUC, 0.765), it did not demonstrate an independent association with PC after adjustment for tumor volume.

 in the context of the small number of carcinoma cases (*n* = 8), which resulted in wide confidence intervals, particularly for sensitivity estimates, and limits the statistical power and stability of multivariable regression models. The high NPVs observed for all evaluated parameters should also be interpreted in the context of the relatively low prevalence of PC in the study cohort.

## Discussion

### Clinical significance of markedly elevated PTH

Parathyroid carcinoma is characterized by markedly elevated PTH levels, which was confirmed in our study. This observation is consistent with the literature, where severe biochemical hyperparathyroidism is considered one of the hallmark features of parathyroid carcinoma [17, 18]. Extremely elevated PTH levels, often accompanied by pronounced hypercalcemia, may therefore raise clinical suspicion of malignancy, although they are not specific enough to establish a definitive diagnosis on their own.

In clinical practice, PTH levels reaching 3–15 times the upper limit of normal are considered highly suggestive of the disease [13, 18, 19, 20], and values exceeding 500 pg/mL (approximately 10 times the normal range of 15–65 pg/mL) are frequently regarded as an important indicator raising suspicion of PC. Patients with parathyroid carcinoma also typically present with serum calcium levels exceeding 3.5 mmol/L (14 mg/dL), a finding reported in approximately 65–75% of cases [21, 22]. In our cohort, the mean serum calcium levels remained lower than those reported above and showed only minimal differences compared to those observed in the benign group, which may be explained by pharmacological management of hypercalcemia prior to surgical admission. This preoperative optimization may partially normalize calcium concentrations and reduce their diagnostic utility.

### Role of tumor volume

Ultrasound is the most commonly used imaging modality due to its non-invasive nature, wide availability, and low cost. Reeder et al. reported that 93% of histologically proven parathyroid adenomas were correctly identified by sonography, with a 100% detection rate for larger adenomas (≥ 1 cm) using grayscale imaging alone. The detection of smaller adenomas often required a combination of grayscale imaging, color and power Doppler, and graded compression techniques [23]. Although ultrasound cannot reliably distinguish benign from malignant parathyroid lesions, certain sonographic features may raise suspicion of malignancy and contribute to the overall clinical assessment [24].

In our cohort, parathyroid carcinomas were characterized by substantially larger tumor volumes compared with benign lesions, further supporting the observation that malignant lesions tend to present as larger masses. Larger tumor size has been consistently reported in the literature as a feature suggestive of malignancy, although considerable overlap with large benign adenomas limits its specificity when used in isolation. Several studies have identified tumor size as an important factor raising suspicion for malignancy [14, 25]. Nevertheless, when interpreted in conjunction with biochemical parameters, tumor volume may contribute to a more comprehensive preoperative assessment. Our findings support the concept that increased tumor mass, particularly when accompanied by biochemical abnormalities, should raise suspicion for parathyroid carcinoma.

### Concept of reduced secretory efficiency

A key finding of our study is the significantly lower PTH-to-tumor-volume ratio in parathyroid carcinoma compared with benign lesions, suggesting reduced secretory efficiency in malignant tumors. We hypothesize that, in parathyroid carcinoma, these regulatory mechanisms are disrupted. Malignant tumors often exhibit functional and structural abnormalities with heterogeneous cellular architecture, which may contribute to impaired hormone secretion and reduced functional efficiency per unit of tumor volume. These features reduce the proportion of functionally active hormone-secreting cells within the tumor. Furthermore, the observed findings may also be interpreted in the context of tumor burden. The higher absolute PTH levels seen in parathyroid carcinoma are likely a consequence of larger tumor size; however, when adjusted for tumor volume, the secretory output appears relatively diminished compared with benign adenomas. This suggests that malignant lesions produce more hormone in absolute terms primarily due to increased mass, rather than higher functional efficiency at the cellular level. In contrast, benign adenomas typically maintain higher metabolic and secretory activity per unit of tumor mass, resulting in a higher PTH output per unit of tissue. From a comparative perspective, parathyroid adenomas are generally composed of more homogeneous, well-functioning chief cells with preserved secretory mechanisms, leading to efficient PTH production relative to their size, whereas carcinomas demonstrate disorganized tissue architecture, areas of non-functional tissue, and impaired cellular coordination, all of which contribute to reduced hormonal efficiency despite larger overall tumor mass [26].

**Fig. 4:**
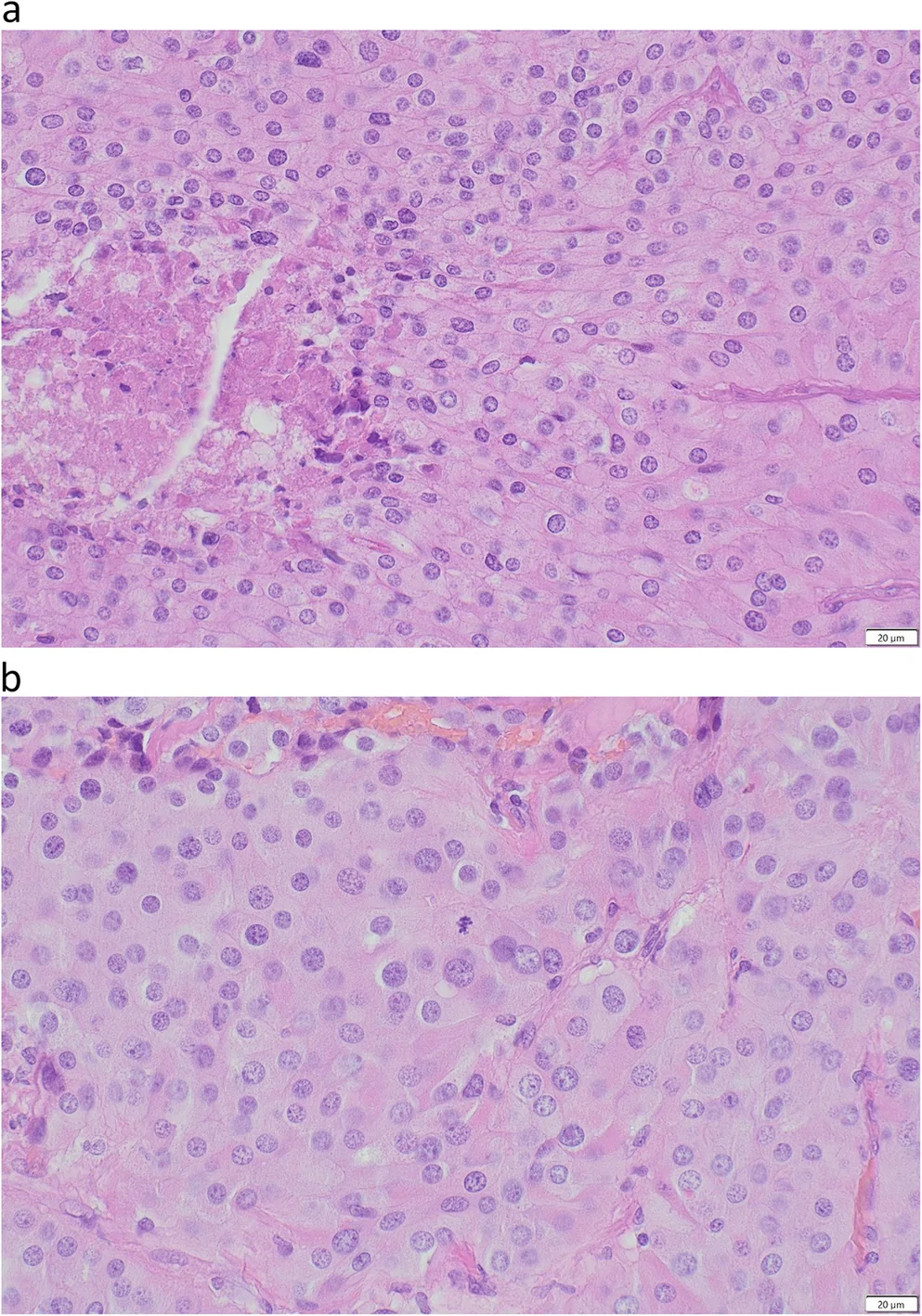
Representative histopathological features of parathyroid carcinoma. **A** Tumor necrosis within parathyroid carcinoma. **B** Increased mitotic activity with identifiable mitotic figures. Hematoxylin and eosin staining; original magnification ×400

This discrepancy may reflect underlying biological differences, including cellular dedifferentiation, loss of normal regulatory mechanisms, and impaired endocrine function within malignant tumors. These mechanisms are supported by pathological and molecular observations. Parathyroid carcinomas frequently demonstrate heterogeneous architecture, fibrosis, and necrosis—features that are uncommon in benign adenomas and may reduce the pool of hormonally active cells, as illustrated in Fig. 4A, B [14]. Additionally, inactivating mutations in the CDC73 gene, leading to loss of parafibromin expression, are present in a substantial proportion of cases and are strongly associated with malignant transformation and altered cellular function [26, 27].

### Preoperative risk stratification

Given the well-recognized difficulty in preoperative differentiation between benign and malignant parathyroid lesions, numerous attempts have been made to develop diagnostic algorithms incorporating clinical, biochemical, and pathological parameters. Proposed models include combinations of serum calcium, intact PTH levels, and patient age, with defined risk thresholds intended to guide surgical decision-making and justify more extensive procedures such as en bloc resection when malignancy is suspected [28]. Similarly, various histopathological and molecular markers, including Ki-67 proliferation index, parafibromin loss, and E-cadherin expression, have been investigated to improve diagnostic accuracy [29, 30]. However, these markers often demonstrate limited sensitivity, overlap between benign and malignant lesions—particularly in atypical adenomas—and are typically available only postoperatively, which restricts their utility in preoperative planning. In this context, the search for reliable preoperative markers remains crucial, as early identification of parathyroid carcinoma directly influences the extent of the initial surgical procedure and, consequently, patient outcomes. Our findings suggest that the PTH-to-tumor-volume ratio may warrant further investigation as a potential adjunctive parameter for preoperative risk assessment. However, its clinical utility remains uncertain and requires validation in larger, multicenter cohorts.

### Clinical implications for surgery

The potential to identify parathyroid carcinoma preoperatively has important clinical implications, particularly for surgical planning. Radical surgery is considered the only effective and potentially curative treatment for parathyroid carcinoma. Complete tumor resection performed during the initial operation remains the gold standard of management [31]. The recommended surgical approach typically involves en bloc resection, including removal of the tumor together with the ipsilateral thyroid lobe and, when indicated, regional lymph nodes and adjacent involved structures. The goal of surgery is to achieve complete tumor removal with negative margins, as the extent and adequacy of the primary surgical procedure are important predictors of prognosis [4, 6, 32]. In contrast, limited parathyroidectomy or simple local excision has been associated with significantly worse outcomes. Several studies have demonstrated that local excision alone, compared with en bloc resection or surgery combined with systematic lymphadenectomy, is associated with a markedly higher risk of local recurrence and disease-related mortality [7, 33, 34]. Previous analyses have also shown that patients treated with oncological resection rather than simple excision experience significantly improved disease-free survival, although these observations were based on relatively limited follow-up periods.

Nevertheless, the available evidence remains somewhat inconsistent. While many reports support the benefit of extensive primary surgery, some studies have not demonstrated a clear correlation between radical resection and improved overall survival. These discrepancies may reflect the rarity of parathyroid carcinoma, small study cohorts, and heterogeneity in surgical management and follow-up. Despite these limitations, most experts agree that complete en bloc resection during the primary operation provides the best chance for long-term disease control and remains the preferred surgical strategy when parathyroid carcinoma is suspected [35].

### Limitations of the study

Several limitations of this study should be acknowledged. First, its retrospective design may introduce inherent biases related to data collection and patient selection. Second, the relatively small number of parathyroid carcinoma cases reflects the rarity of the disease and limits the statistical power of subgroup analyses. Third, the study was conducted in a single center, which may affect the generalizability of the results. Additionally, tumor volume was estimated using preoperative ultrasound, which is operator-dependent and may introduce measurement variability.

### Future directions

Future research should focus on prospective, multicenter studies to validate the findings of the present analysis. In particular, external validation of the PTH-to-tumor-volume ratio as a diagnostic marker is warranted. Establishing standardized cutoff values, including the identification of an optimal threshold (cut-off point), may further enhance its clinical utility in differentiating parathyroid carcinoma from benign parathyroid lesions. Integration of biochemical, imaging, and potentially molecular markers could ultimately improve preoperative risk stratification and guide optimal surgical management.

## Conclusions

In this retrospective, single-center cohort, parathyroid carcinomas were associated with lower PTH-to-tumor-volume ratios than benign parathyroid lesions. This exploratory finding requires validation in larger, multicenter cohorts before its diagnostic and prognostic value can be established. At present, the PTH-to-tumor-volume ratio should not be used as a standalone parameter for clinical decision-making.

## Abbreviations

AUC: Area under the curve
CDC73: Cell division cycle 73
CI: Confidence interval
FNAB: Fine needle aspiration biopsy
IQR: Interquartile range
MEN: Multiple endocrine neoplasia
NPV: Negative predictive value
OR: Odds ratio
PC: Parathyroid carcinoma
PHPT: Primary hyperparathyroidism
PTH: Parathyroid hormone
PPV: Positive predictive value
ROC: Receiver operating characteristic
SD: Standard deviation
SPSS: Statistical Package for the Social Sciences
WHO: World Health Organization
